# From discovery to innovation in physiological research

**DOI:** 10.1113/EP092125

**Published:** 2024-10-28

**Authors:** Morten Zacho

**Affiliations:** ^1^ Department of Sports Science and Clinical Biomechanics University of Southern Denmark Odense Denmark

In 1953, Watson and Crick discovered the structure of DNA (Watson & Crick, 1953). Fifty years later, in 2003, the human genome project was completed, with ∼92% of the human genome sequenced (Collins et al., 2003). Between these two landmark achievements in science lie numerous smaller discoveries and innovations. One of the key events was the development of the polymerase chain reaction (PCR) technique (Saiki et al., 1985). The basic principles were described in a paper from 1971 (Kleppe et al., 1971), but the breakthrough development is attributed to Kary Mullis. The story goes that Mullis conceived the idea of using heat cycles to amplify DNA while driving one evening in the mountains of California. His thoughts wandered, and he visualized the movement of man‐sized molecules; a classic eureka moment (Mullis, 1990). However, the method proved effective only after numerous adjustments by his colleagues and the application of Taq polymerase isolated from the bacterium *Thermus aquaticus* (Saiki et al., 1988). The bacterium was first identified in hot springs in Yellowstone National Park in 1969 by Thomas D. Brock (Chien et al., 1976), naturally without knowing how much importance it would later have. Finally, the increasing use of PCR in laboratories worldwide was largely attributable to the development of the commercial thermocycler. A fascinating aspect of this story is the mix of discoveries, ideas and innovations that together form the potential for groundbreaking research. The discoveries of DNA structure and Taq polymerase, along with the radical, innovative idea of PCR, were key milestones. Ultimately, it was the incremental innovation of the thermocycler that enabled large‐scale analyses, culminating in the sequencing of the human genome.

It is important to recognize that a single idea or invention does not, on its own, constitute innovation. Innovation occurs only when an idea or invention is developed and implemented in a way that fundamentally changes how we do things. For example, the light bulb was invented 40 years before Thomas Edison made it relevant for general use. Or consider PCR, where the principal idea was conceived 15 years before Mullis and colleagues made the method applicable to practical use.

Innovation comes in many forms (Table 1). Radical innovation and incremental innovation are well‐known examples, but it is also relevant to consider perspectives of architectural innovation in research. This type of innovation is not about physical architecture but refers to changing the framework or infrastructure around a product or concept (Henderson & Clark, 1990). The core aspects of the product remain the same, but the application or context is transformed. The value of considering architectural innovation is that it does not rely on new inventions but rather on repurposing concepts from other existing fields. A well‐known example of this is Uber. There is nothing novel about taxi driving, but integrating it into an app‐based service model represents significant innovation. Uber is an example not only of architectural innovation but also of disruptive innovation, because Uber caused massive disturbances in the established taxi industry. In research, an example of architectural innovation could be continuous blood glucose monitoring. Although the technology has existed for decades, recent advances in the portability of these devices now allow for large‐scale field studies with 24/7 data collection (Lee et al., 2021).

**TABLE 1:** Overview of different concepts related to innovation.

Concept	Description	Example
Discovery	Uncovering something that already exists	The structure of DNA
Idea/invention	Possible starting point for innovation, although many ideas and inventions are never materialized	The idea and invention of PCR
Incremental innovation	Small improvements to an existing concept	The gradual development of the thermocycler
Radical innovation	Implementation of an idea that substantially introduces a new way of doing things	PCR in combination with the automated thermocycler
Disruptive innovation	Radical innovation that fundamentally alters an existing field	PCR transformed the field of bioscience
Architectural innovation	A known concept applied in a new context or infrastructure	Using continuous blood glucose monitoring in large field studies

The distinctions between different types of innovations are not rigid, and most examples will embody characteristics from multiple categories. Moreover, various other terms are used to describe innovation in different fields. Nevertheless, recognizing the traits of different innovation types is valuable when planning and executing innovation strategies, helping to avoid an exclusive focus on the challenging radical innovation. Prioritizing incremental innovation can drive steady progress year after year. Regardless of the type of innovation in focus, it is crucial that innovative thinking permeates all levels of an organization, because it often involves questioning the established and exploring paths that may lead to dead ends. A key element in fostering innovation is encouraging divergent thinking, exploring a broad range of possibilities, considering unconventional approaches and looking beyond the obvious. Although convergent thinking is also essential, it is the dynamic interplay between the two that drives new ideas.

Pursuing innovation is not only a matter of new ideas from eureka moments. It is very much a systematic search for opportunity gaps in the procedures with which we work. Besides ideas and optimizations, opportunity gaps can be found by identifying pain points. Pain points are typically described as ‘the problems, frustrations or challenges that customers face when they interact with your product or service’. In research, pain points might relate to unattainable results, gaps in methods or knowledge, or obstacles to overcome. Prioritizing time to identify and address pain points methodically with a cross‐disciplinary team can yield surprisingly good results.

A prime example of a company with innovation at its core is SpaceX. The need for innovation arises from the simple fact that to accomplish something unprecedented (something for which the necessary technology does not yet exist), the company must be extraordinarily innovative. The goal of sending hundreds of Starships to Mars requires this mindset. To embed innovative thinking into the company, SpaceX has formulated what they call an ‘innovation algorithm’, a strategy designed to promote optimizations and new ideas that can accelerate progress (Running the Algorithm: SpaceX's Approach to Exponential Growth, www.youtube.com/watch?v=ZOWakxXjotg). Although not all of SpaceX's principles may be applicable directly to research, parallels can be drawn between sequencing the human genome and sending rockets to Mars.

Two aspects of the approach taken by SpaceX can be particularly relevant for research. The first is the notion of rethinking constraints and requirements: ‘Make the requirements less dumb. Challenge constraints and requirements.’ Every research project is subject to numerous requirements and regulations, from ethical guidelines to specific procedural steps in the laboratory. Although it is essential to follow rules, some constraints might stem from outdated contexts, with their original rationale forgotten or overlooked. Re‐examining these requirements can sometimes reveal opportunities for improvement.

The second principle is: ‘Delete the part or process step’, also known as: ‘The best part is no part.’ Researchers often aim to incorporate as many data collection streams and analyses as possible. However, the more we add, the greater the risk of reducing efficiency and increasing errors. To achieve the key objectives within the available time frame and budget, it is crucial to eliminate unnecessary steps. Going back to the development of PCR before the application of Taq polymerase, researchers had to add new enzyme at each step because the thermosensitive enzyme was destroyed in the heat cycle. That was extremely time consuming and probably frustrating. Being able to delete that step changed the usability of PCR.

The potential of a systematic approach to innovation in research lies both in optimizing processes and in achieving landmark results. Looking at fields outside physiological research, much innovation is currently catalysed using artificial intelligence (AI) and large language models (LLMs) (Mariani et al., 2023). AI and LLMs have obvious applications for optimizing communication, extracting information, writing code, etc., but the impressive performance of these models also warns us to be cautious, because convenience in use might triumph over stringency (Abdurahman et al., 2024). The uncertainty of AI applications is a reason for scepticism but can also be seen as a window for innovation, because the full potential as a research tool is by no means clear. In a recent review by Wang & Chen (2025), they cover how AI and LLMs are being applied to neuroscience and how this might potentially reshape the landscapes of neuroscience research. Although the extent to which AI will drive innovation in physiological research remains uncertain, AI is a good example of looking outside one's own field for opportunity spaces with the right blend of potential and challenges.

In conclusion, innovation can stem from a new idea (or even an old idea), but its true value lies in implementing that idea in a way that profoundly impacts how we operate. To foster innovation effectively, it must be supported by leadership and embedded into the organizational culture. This cultural foundation includes a willingness to challenge established norms and accept the inevitable pursuit of ideas that may not succeed. It also requires a balanced approach that embraces divergent thinking, rather than relying solely on convergent, analytical thought. As in commercial industries, an optimized innovation strategy in research has the potential to accelerate the rate at which we generate new knowledge and make significant discoveries.

## AUTHOR CONTRIBUTIONS

Sole author.

## CONFLICT OF INTEREST

None declared.

## FUNDING INFORMATION

None.
